# The Role of Pumilio RNA Binding Protein in Plants

**DOI:** 10.3390/biom11121851

**Published:** 2021-12-09

**Authors:** Sung Un Huh

**Affiliations:** Department of Biology, Kunsan National University, Gunsan 54150, Korea; sungun@kunsan.ac.kr; Tel.: +82-63-469-4587; Fax: +82-63-469-7421

**Keywords:** RNA binding protein, Pumilio, translational modification, RNA biomolecules

## Abstract

Eukaryotic organisms have a posttranscriptional/translational regulation system for the control of translational efficiency. RNA binding proteins (RBPs) have been known to control target genes. One type of protein, Pumilio (Pum)/Puf family RNA binding proteins, show a specific binding of 3′ untranslational region (3′ UTR) of target mRNA and function as a post-transcriptional/translational regulator in eukaryotic cells. Plant Pum protein is involved in development and biotic/abiotic stresses. Interestingly, Arabidopsis Pum can control target genes in a sequence-specific manner and rRNA processing in a sequence-nonspecific manner. As shown in in silico *Pum* gene expression analysis, Arabidopsis and rice *Pum* genes are responsive to biotic/abiotic stresses. Plant Pum can commonly contribute to host gene regulation at the post-transcriptional/translational step, as can mammalian Pum. However, the function of plant Pum proteins is not yet fully known. In this review, we briefly summarize the function of plant Pum in defense, development, and environmental responses via recent research and bioinformatics data.

## 1. Evolutionally Conserved Pumilio RNA Binding Proteins

Sequence-specific RNA binding proteins (RBPs) could play important roles in transcriptional/translational regulation via direct binding to specific *cis*-acting elements of target mRNA, usually located in 3′-untranslated regions (UTRs) [1,2,3]. Furthermore, most known RBPs might repress translation by binding to a 3′ UTR because they contain regulatory elements for mRNA stability, localization and translation [4,5,6]. Other RBPs also can affect translation regulation in 5′ UTRs because of their ribosomal protein binding site [7]. Sometimes, RBPs work with miRNAs to control target mRNAs and to mediate the cell-to-cell trafficking of RNAi signals [8,9].

**Figure 1:**
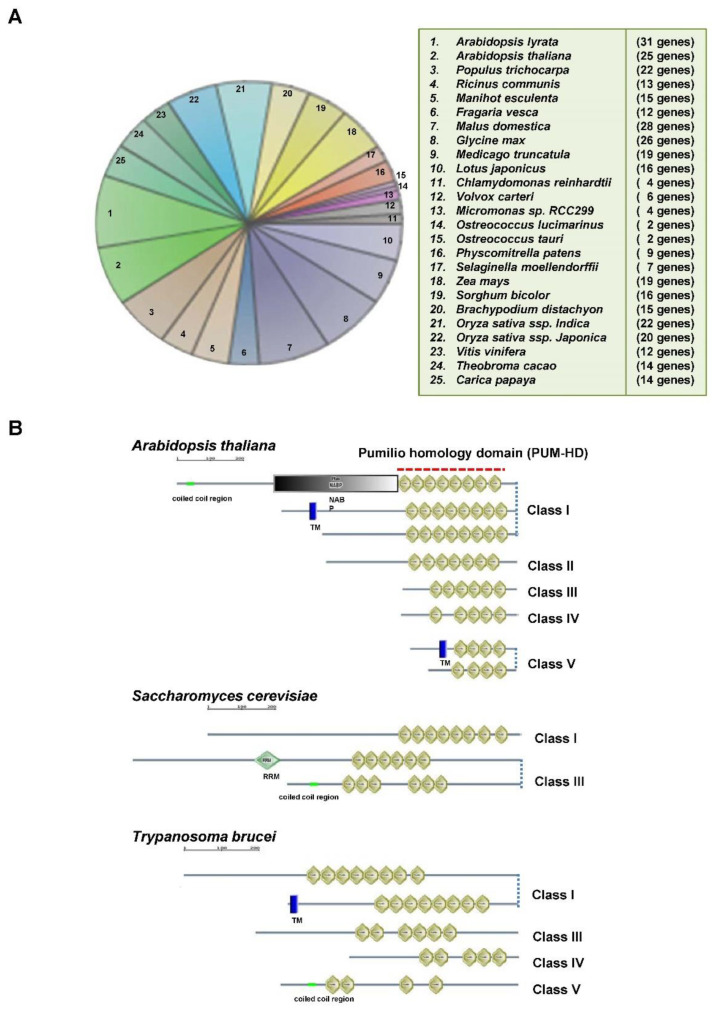
Analysis of putative *Pum* in green plant species. (**A**) Pum-HD is used in the analysis of putative plant *Pum* genes. The functional clusters are associated with InterPro domain (IPR001313) via Plaza 2.5, which is a comparative genomics analysis tool (http://bioinformatics.psb.ugent.be/plaza/ accessed on 13 November 2021). (**B**) Classified via alpha helical repeats of Pum-HD. *Arabidopsis thaliana*, *Saccharomyces cerevisiae* and *Trypanosoma brucei* Pum proteins are analyzed via SMART tool (http://smart.embl-heidelberg.de/ accessed on 13 November 2021). The Pum class is divided into the numbers of alpha helical repeats of Pum-HD. Class I contains eight alpha helical repeats of Pum-HD. Class II contains seven alpha helical repeats of Pum-HD. Class III contains six alpha helical repeats of Pum-HD. Class IV contains five alpha helical repeats of Pum-HD. ClassV contains four alpha helical repeats of Pim-HD. Other domains are indicated in the box.

Pumilio (Pum)/Puf family RNA binding proteins are highly conserved in various organisms [10,11]. Interestingly, plant *Pum* genes have more highly diverse members than mammalian *Pum* genes. For instance, human and mouse genes have the two homologs [11,12,13]. *Caenorhabditis elegans* and *Saccharomyces cerevisiae* have eleven and six homologs, respectively [14,15]. *Trypanosoma cruzi* and *Plasmodium falciparum* have ten and two homologs, respectively [16,17]. *Drosophila melanogaster* encodes two functional protein isoforms. These Pums are implicated in diverse physiological processes, specifically development [18]. When affinity-tagged-Pum is expressed in the ovaries of *D. melanogastor*, putative Pum-associated mRNAs were recovered in over 1000 genes which are believed to contain Pumilio RNA-binding motifs at the 3′ UTRs [19]. It might be possible that just one Pum could control about 10% of all genes in *D. melanogaster.* Similarly, yeast Pum proteins have multiple RNA targets [14]. Thus, it is important to understand translational regulations via Pum proteins because of their specific capacity to bind to *cis*-elements in the 3′ UTR of target mRNA. In the present study, plant Pums showed that the Arabidopsis, rice, soybean, apple, and corn genomes contained the largest number of putative Pum homolog proteins, about 20 homologous members, via analysis with Plaza 2.5, which is a comparative genomics analysis tool (Figure 1A) [20]. On the contrary, green algae such as *Ostreococcus tauri*, *Chlamydomonas reinhardtii* and *Volvox carteri* have small numbers of putative *Pum* genes in their genomes (Figure 1A). It is reasonable that plants have larger genomes than other eukaryotic organisms. In a plant’s evolution, it might have developed mechanisms to cope with abiotic and biotic stresses imposed by the adverse environment and pathogen infections. Thus, a plant might need to have a complicated regulation system against adverse conditions. The diversity of plant *Pum* genes could contribute to the post-transcriptional/translational regulation of many genes such as identified mammalian Pums.

## 2. RNA-Binding Specificity of Pumilio-Homology Domain

To date Pum proteins have been described to be defined by a highly conserved Pumilio RNA-binding domain in the C-terminal region. Pum classes were sorted by the numbers of the alpha helical repeats of the Pumilio-homology domain (Pum-HD) via SMART tool and were mainly represented as the canonical and non-canonical Pum-HD (Figure 1B) [21]. Pum-HD is necessary and sufficient for sequence-specific RNA binding [22,23,24]. Basically, the eight alpha helical repeats of Pum-HD could confer recognition and binding affinity to a Pumilio RNA binding motif of the target RNA [25]. Engineered and customized Pum-HD protein could help control specific target regulation [26,27,28,29,30]. In yeast, PUF3, 4 and 5 have putative targets of about 200 genes, and the target mRNA contains the conserved ‘UGUX_3-5_UA’ motifs [14,31]. Arabidopsis Pum, APUM5 has eight alpha helical repeats and confers resistance to *Cucumber mosaic virus* (CMV) via direct binding to CMV 3′ UTR contained in a putative Pumilio RNA-binding motif. A recombinant GST-APUM5-Pum-HD protein has binding activity with regard to the Nanos regulator element sequence (*NRE*), which is recognized by *Drosophila* Pum [32,33]. APUM1 to 6 members also exhibit binding activity with regard to the *NRE* in yeast three hybrids [34]. Yeast PUF2 has one RNA-recognition motif (RRM) and six alpha helical repeats of Pum-HD (Figure 1B). Yeast PUF1 and PUF2 might posttranscriptionally regulate the cell wall integrity pathway by controlling the *Zeo1* gene in CaCl_2_ stress [35]. However, *ASH1* is the target of yeast PUF6, which contains six alpha helical repeats of Pum-HD [36]. This means that fewer alpha helical repeats of Pum-HD can confer the binding activity of target RNA, but it is hard to identify the regulation of target RNA.

The function of APUM23, which contains seven alpha helical repeats of Pum-HD, is involved in pre-rRNA processing in the nucleolus. The *apum23* mutant exhibits a similar developmental phenotype to nucleolin and ribosomal protein mutants [37]. Similarly, *S.* *cerevisiae* PUF4, which contains eight alpha helical repeats of Pum-HD, binds to mRNAs encoding nucleolar ribosomal subunits [14,25]. This indicates that plant Pum protein could possibly contribute to the regulation of pre-RNA processing via target binding of nucleolar ribosomal RNA-processing factors.

Some plant Pum proteins contain another functional domain. For example, the nucleic-acid binding protein (NABP) domain has nucleic acid binding activity [38]. It is believed to bind to RNA with a broad specificity and is especially found in several plant Pum-HDs (Figure 1B). The NABP domain is specific to the plant Pum family, and its function is not clear. RNA RRM is one of the most abundant RBDs in eukaryotic cells and confers a variety of RNA bindings [39]. Several plant Pums contain an RRM. It might function in RNA recognition as a helper protein (Figure 1B) [6]. Additionally, plant Pums have a transmembrane domain which has a single transmembrane alpha helix and is specifically localized to membrane compartments (Figure 1B) [40].

## 3. Protein–Protein Interaction in Plant Pum

Pum-HD has a dual function. The inner surface of Pum-HD binds to the conserved ‘UGUX_3-5_UA’ motif in the 3′ UTR regions of the target gene. Interestingly, the outer surface of Pum-HD also permits protein–protein interactions with diverse proteins [6,41]. The yeast Pum protein, PUF5p, binds to and represses *HO* mRNA, which encodes a DNA endonuclease via binding to the Pumilio-RNA binding motif. Pop2p, which encodes a component of the Ccr4p-Pop2p-Not deadenylase complex, is required for PUF5p-mediated *HO* mRNA decay [42]. It affects both mRNA stability and translational efficiency [43,44]. Thus, Pums do not work alone to repress the target mRNA. Furthermore, yeast PUF5p and Pop2p interaction is evolutionally conserved in *C*. *elegans* PUF8 and *H*. *sapiens* (PUM1) [42]. Arabidopsis Pum, APUM5, also interacts with plant Ccr4 homologues [45]. Sometimes, yeast PUF5p does not require Ccr4-Pop2 deadenylase when PUF5p responds to DNA replication stress [46]. Thus, plant Pum function could be associated with deadenylase-dependent and -independent pathways via formation, as with Ccr4-Pop2-NOT mRNA deadenylase complexes. 

Human PUM2 can make a complex with Argonaute (Ago) miRNA-binding protein, and then, the PUM2-Ago heterodimer is associated with the core translational elongation factor (eEF1A). This complex inhibits eEF1A GTPase activity and translational elongation [47]. These results imply that plant Pum could be associated with the Ago-eEF1A protein complex.

## 4. Dynamic Subcellular Localization of Plant Pum

To regulate diverse target genes, Pum might have to locate in other organelles or some specific regions at the subcellular level. Plant Pum has various subcellular localization patterns [37,40,48]. For example, APUM9 has a transmembrane domain in the N-terminal region and locates the cytoplasmic-punctuated structure region [48]. APUM5 has a putative transmembrane domain in the N-terminal region and localizes to cytoplasmic-punctuated structures and vacuolar structures in tobacco epidermal cells [40]. Recently, regarding APUM6 subcellular localization, it has been clearly observed that APUM6 localizes on the surface of the endoplasmic reticulum (ER) but still shows dynamic localization patterns [10]. APUM6 might have diverse functions in different organelles. APUM23 and APUM24 have a putative nuclear localization signal in the C-terminal region and normally localize to nucleolar-like structures in planta [37,48,49]. Interestingly, ChPUM2 and ChPUM3 from *Chara corallina* also show similar nucleolar localization [49].

## 5. The Role of Pum in Plant Development

**Figure 2:**
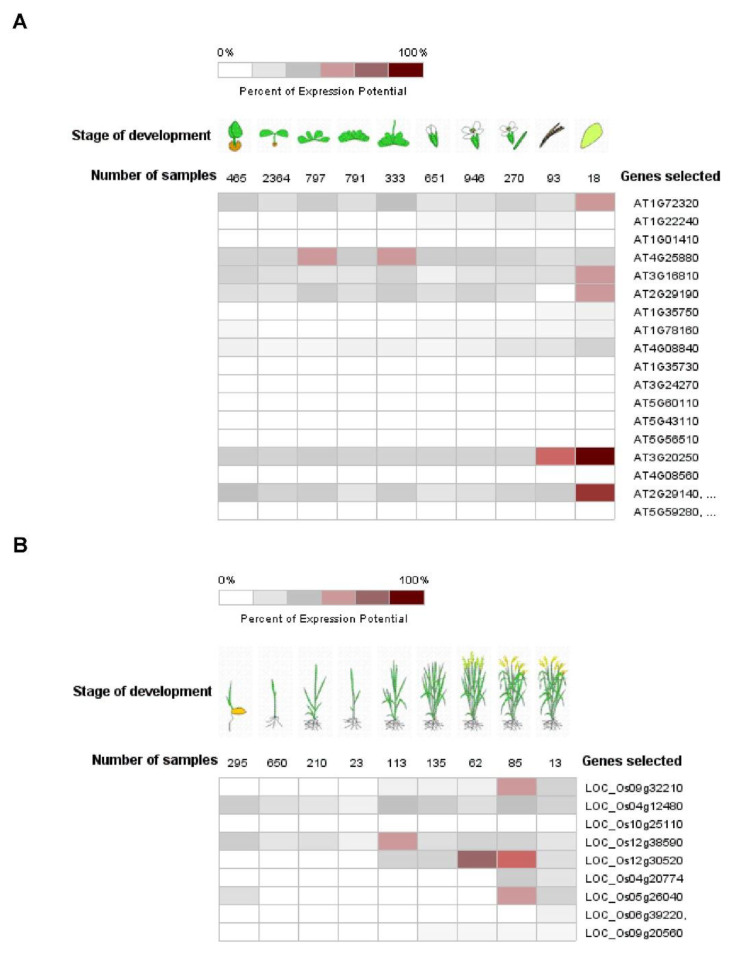
Expression pattern of Arabidopsis and rice *Pum* in development. (**A**,**B**) Expression pattern of Arabidopsis and rice *Pum* was analyzed at developmental stage using the Genevestigator (https://www.genevestigator.com accessed on 13 November 2021). Expression levels of Arabidopsis and rice *Pum* vary under developmental stages. Transcript levels were assessed using microarray data available from Genevestigator.

**Figure 3:**
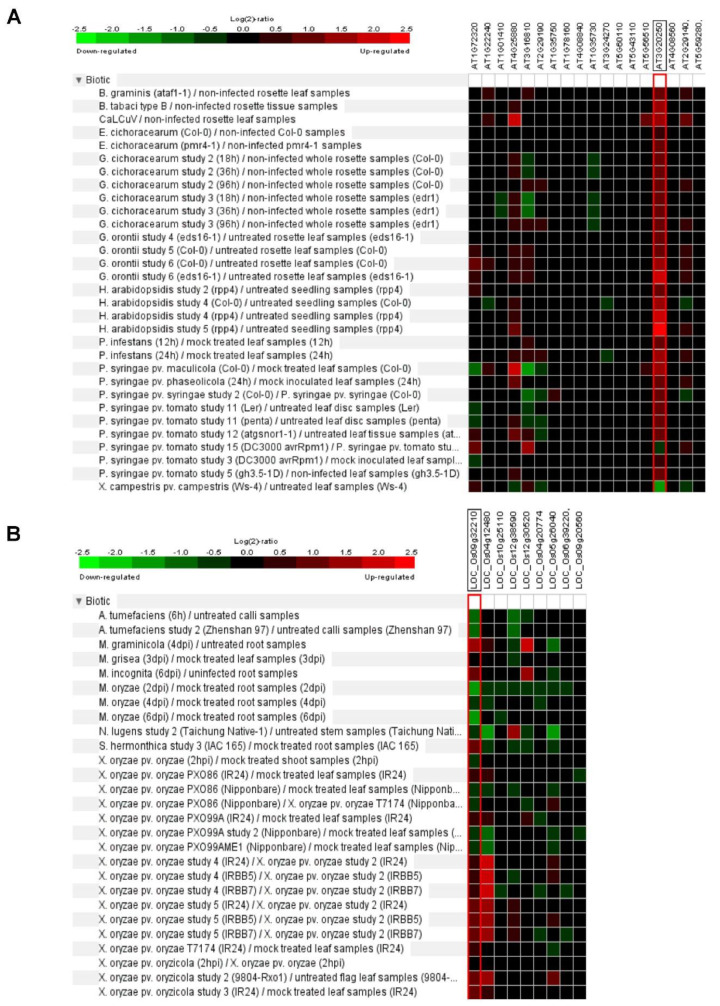
Biotic stress response of Arabidopsis and rice *Pum*. (**A**,**B**) Expression pattern of Arabidopsis and rice *Pum* is analyzed in biotic stress response using the Genevestigator. Arabidopsis and rice *Pum* transcript levels were assessed using microarray data available from Genevestigator. Expressions patterns were searched in a range of biotic stress conditions in this database. Red colors indicate genes that were upregulated relative to the control for a given treatment, and green colors indicate genes that were downregulated relative to the control.

Pum proteins play important roles during development, differentiation and cell cycle regulation in various organisms [33,50]. APUM1 to APUM6 are specifically associated with shoot stem cell maintenance genes such as *WUSCHEL* (*WUS*) and *CLAVATA1* (*CLV1*) [34,51,52]. Like the mammalian system, human PUM2 is expressed in embryonic stem cells and could affect germ cell development [53]. Mammalian PUM1 and PUM2 regulate cell cycle inhibitor Cdkn1b via translational control [54]. *C*. *elegans* Pum, PUF8, might function as a repressor of the stem cell proliferative fate via control of GLP-1/Notch signaling in germline cells [55]. Likewise, plant Pum could affect a variety of development stages. Gene expressions of *APUM23* and *APUM24* were shown to be continuously expressed in all the developmental stages and were enhanced at the seed stage (Figure 2A). APUM23 affects pre-ribosomal RNA processing in the nucleolus and the *apum23* mutant showed a developmental defeat phenotype [37]. It is possible that APUM24 could function in the ribosomal RNA processing because APUM24 predominantly localizes to the nucleolus [49]. *APUM5* and *APUM6* were highly abundant at developmental stages (Figure 3). However, these mutants did not show abnormal developmental phenotypes. APUM1 to APUM6 have a similar protein structure and might have the same redundancy. In the future, this will be studied for genetic crossover with *APUM1* to *APUM6* mutants. In rice, at least four rice *Pum* genes are highly expressed at the vegetative stage (Figure 2B). Plant Pum proteins could play a role in development and differentiation.

## 6. Plant Pum Function in Biotic Stress

In Arabidopsis, some putative target RNAs of Pum were identified by yeast in a three-hybrid system. These genes contained Pumilio RNA binding motifs at the 3′ UTR regions [34]. For example, *Responsive to dehydration 19* (*RD19*) and *Plant homeodomain* (*PHD*) containing protein (At3g63500) were isolated [34]. RD19 encodes cysteine protease and is involved in resistance and gene-mediated immunity via interaction with *Ralstonia solanacearum* type III effector PopP2 [56]. This means that plant Pum could be related to plant immunity. APUM5 is involved in plant defense against CMV infection and function as a translational repressor via binding to the 3′ UTR of CMV [32]. Furthermore, gene expression analysis of Arabidopsis *Pum* family exhibits enhanced or repressed expression levels upon the *Cabbage leaf curl virus* (CaLCuV), *Golovinomyces cichoracearum*, *Hyaloperonospora arabidopsidis*, *Peudomonas syringae* and *Xanthomonas campestris* (Figure 3A). Similarly, rice *Pum* genes are upregulated or downregulated by *Mycosphaerella graminicola*, *Meloidogyne incognita*, *Magnaporthe oryzae*, *Nilaparvata lugens* and *Xanthomonas oryzae* infections (Figure 3B). When infected with *M. oryzae,* most of the rice *Pum* genes were shown to be repressed. Expression levels of *OS09g32210* and *OS04g12480* genes were highly enhanced upon *X. oryzae* infection in IRBB5/7 (resistance) and IR24 (susceptible) rice plants (Figure 3B). Thus, infectious pathogens may affect plant *Pum* gene expression and attenuate plant immunity. In *D. melanogaster Pum* mutants, some antibacterial genes are highly expressed [19]. In particular, CG18372 (Attactin-B), CG4740 (Attactin-C), CG7629 (Attactin-D), CG1373 (Cecropin C) and CG13422 (Gram-negative binding protein) were encoded antibacterial peptides [19]. The immunity function of Pum in plant and mammalian genes will be important to investigate in the extended study of transcriptional/translational control.

## 7. Plant Pum Function in Abiotic Stress

**Figure 4:**
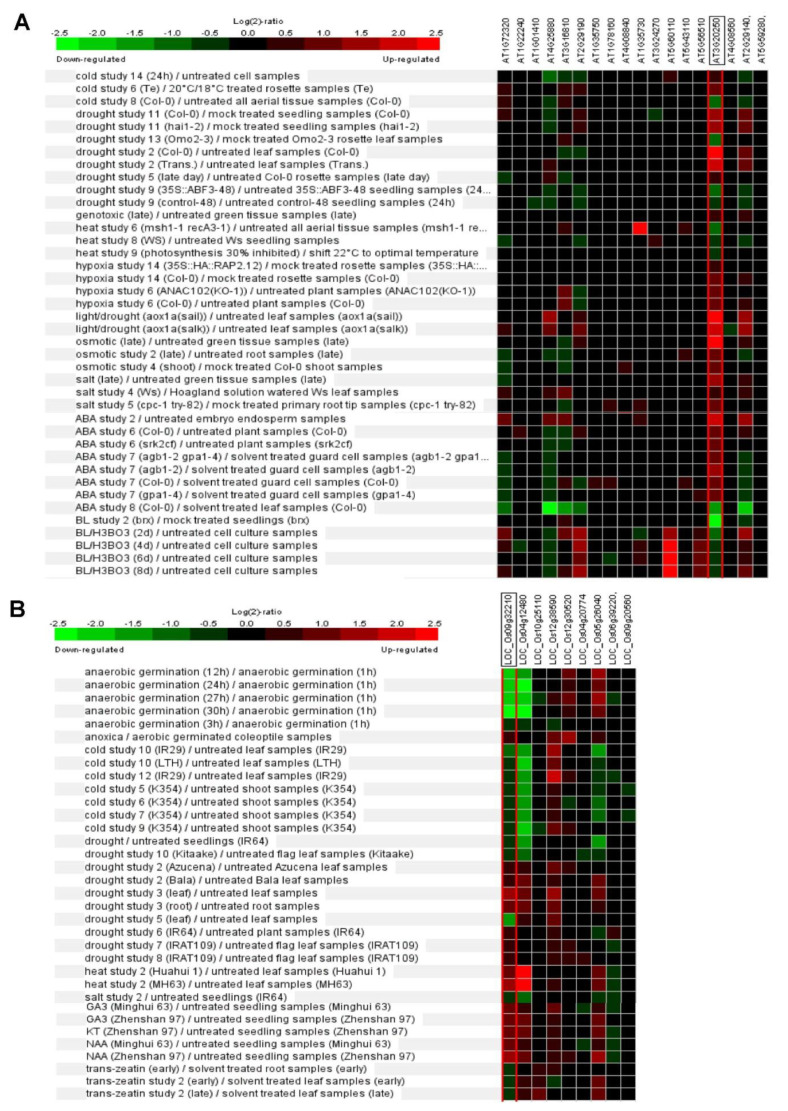
Differential expression patterns of *Pum* genes in Arabidopsis and rice in response to abiotic stresses or phytohormones. (**A**) Expression pattern of Arabidopsis *Pum* was analyzed in abiotic stresses (cold, drought, heat, hypoxia, osmotic and salt) and phytohormones (ABA and brassinosteroid) treatments using Genevestigator. (**B**) Expression pattern of rice *Pum* was analyzed in abiotic stresses (anaerobic, cold, drought, heat, and salt) and phytohormones (gibberellin, auxin, and cytokinin) treatments using Genevestigator. Arabidopsis and rice *Pum* transcript levels were assessed using microarray data available from Genevestigator. Expressions patterns were searched in a range of abiotic stress conditions in this database. Red colors indicate genes that were upregulated relative to the control for a given treatment, and green colors indicate genes that were downregulated relative to the control.

Phytohormone abscisic acid (ABA) and brassinosteroid (BR) have essential roles in the manipulation of abiotic stress responses [57,58]. Expression patterns of Arabidopsis *Pum* are dynamically changed by ABA and BR treatment (Figure 4A). Recently, the *apum23* mutant exhibited altered gene expression patterns of ABA and salt-stress-responsive genes. APUM23 is essential for salt sensitivity in response salt stress [59]. APUM5 has also been reported to be involved in salt tolerance [60]. The overexpression of *APUM9* results in enhanced heat stress tolerance [61]. Furthermore, the expression of several Arabidopsis *Pum* genes is changed by environment stresses such as cold, drought and salt stresses (Figure 4A). Rice *Pum* genes responded to auxin and cytokinin but not ABA (Figure 4B). Basically, cytokinin and auxin are also deeply associated with abiotic stress responses [62,63]. Plant Pum can influence the target RNA through environmental stresses and phytohormone molecules.

## 8. The Multifunctional Plant Pum Protein and Crop Engineering

The translational control of gene expression is an important step for the survival of plants. Pum proteins evolutionally conserve the canonical Pum-HD RNA binding domain in most of the eukaryotes. This Pum-HD confers target RNA specificity via direct binding and functions as a protein–protein interaction platform. Sometimes, Pum acts as a translational activator. Until now, plant Pum functions were not well known. The plant *Pum* family contains the highest number of members, about twenty, compared with the mammalian *Pum* family. This implies that many plant Pum proteins might have plant-specific functions and could have plant-specific targets. From the in silico data analysis, it is shown that plant Pums could be associated with a variety of signaling pathways. This regulation could be more complicated because plant hormones exhibit crosstalk between different plant hormones. A better understanding of the plant Pum regulation mechanism would help innovate new strategies to improve crop plant engineering. Thus, it would be interesting to identify the putative RNA targets and generate customized Pum-HD proteins for agriculture.

## Data Availability

Not applicable.
